# Targeted Gene Knockouts by Protoplast Transformation in the Moss *Physcomitrella patens*

**DOI:** 10.3389/fgeed.2021.719087

**Published:** 2021-12-17

**Authors:** Lei Zhu

**Affiliations:** Department of Botany and Plant Sciences, University of California, Riverside, CA, United States

**Keywords:** Gene Targeting, Homologous Recombination, Protoplast Transformation, CRISPR-Cas9, Knockouts, *Physcomitrella patens*

## Abstract

Targeted gene knockout is particularly useful for analyzing gene functions in plant growth, signaling, and development. By transforming knockout cassettes consisting of homologous sequences of the target gene into protoplasts, the classical gene targeting method aims to obtain targeted gene replacement, allowing for the characterization of gene functions *in vivo*. The moss *Physcomitrella patens* is a known model organism for a high frequency of homologous recombination and thus harbors a remarkable rate of gene targeting. Other moss features, including easy to culture, dominant haploidy phase, and sequenced genome, make gene targeting prevalent in *Physcomitrella patens*. However, even gene targeting was powerful to generate knockouts, researchers using this method still experienced technical challenges. For example, obtaining a good number of targeted knockouts after protoplast transformation and regeneration disturbed the users. Off-target mutations such as illegitimate random integration mediated by nonhomologous end joining and targeted insertion wherein one junction on-target but the other end off-target is commonly present in the knockouts. Protoplast fusion during transformation and regeneration was also a problem. This review will discuss the advantages and technical challenges of gene targeting. Recently, CRISPR-Cas9 is a revolutionary technology and becoming a hot topic in plant gene editing. In the second part of this review, CRISPR-Cas9 technology will be focused on and compared to gene targeting regarding the practical use in *Physcomitrella patens*. This review presents an updated perspective of the gene targeting and CRISPR-Cas9 techniques to plant biologists who may consider studying gene functions in the model organism *Physcomitrella patens*.

## Introduction

Gene targeting mediated by homologous recombination, widely applied in mouse embryonic stem cells, is particularly useful for studying gene functions. This invention won the Nobel Prize in physiology and medicines in 2007 (Capecchi, 2005a). In plants, the moss *Physcomitrella patens* is the only species harboring high gene targeting efficiency; therefore, gene targeting in *Physcomitrella* has been extensively used (Schaefer and Zryd, 1997). Since the first successful moss knockouts were obtained, thousands of publications emerged from adapting gene targeting to the *Physcomitrella* research (Girke et al., 1998; Rensing et al., 2020).

CRISPR-Cas9 won the Nobel Prize in 2020, is a revolutionary technology, and has become a hot topic in plant and animal research to study gene functions and create variants for breeding (Ran et al., 2013; Hsu et al., 2014; Knott and Doudna, 2018). One advantage of CRISPR-Cas9 is that it can make marker-free knockouts suitable for generating transgene-free plants or animals. In plants, CRISPR-Cas9 has been used in species including model organism Arabidopsis and commercial plants such as tobacco, maize, soybean, and wheat (Jiang et al., 2013; Nekrasov et al., 2013; Shan et al., 2013; Liang et al., 2014). Recently, researchers would like to take advantage of CRISPR-Cas9 to study gene functions in *Physcomitrella patens*.

This review will not cover all the applications of the powerful technologies but rather focus on the practical use of successfully targeted knockouts resulting from traditional gene targeting and CRISPR-Cas9 technology.

## Overview of Gene Targeting in *Physcomitrella Patens*

Targeted gene knockout for generating loss-of-function alleles is essential in genome editing, allowing for the precise characterization of gene functions in cell growth, organism development, and physiological processes. Thus, many genome editing tools have been deployed to obtain knockouts, including nucleases such as zinc-finger nucleases (ZFNs), transcription activator-like effector nucleases (TALENs), RNA-guided CRISPR-Cas family, and gene targeting mediated by endogenous homology-directed repair (HDR) (Capecchi, 2005b; Miller et al., 2007; Sander et al., 2011; Wood et al., 2011; Cong et al., 2013; Knott and Doudna, 2018). In addition, gene targeting utilizes knockout constructs to target genes through homologous recombination (Schaefer, 2001; Ermert et al., 2019).

Gene targeting is widely applied in mice, yeast, and other organisms (Gardner and Jaspersen, 2014; Gerlai, 2016). In the yeast *Saccharomyces cerevisiae*, the predominant mechanism for repairing double-strand break (DSB) is homologous recombination. Thus, yeast exhibits high efficiency of gene targeting. The mouse is another model organism traditionally deployed to study gene knockouts, in which researchers can use precise genome editing tools (Bouabe and Okkenhaug, 2013). However, nonhomologous end joining (NHEJ) is the dominant pathway to repair double-strand breaks in most plants and mammals. In those organisms, foreign DNA fragments are inserted into the genome illegitimately. While most plants exhibit a very low efficiency of homologous recombination (less than 1%), *Physcomitrella patens* is the only plant species exhibiting high efficiency (up to 90%) of homologous recombination, resulting in a high ratio of targeted integration to illegitimate insertions into moss genome (Schaefer and Zryd, 1997; Quatrano et al., 2007). Homologous recombination in *Physcomitrella patens* is dependent on RAD51 to repair DSBs (Markmann-Mulisch et al., 2007; Schaefer et al., 2010). Additionally, many genes have been functional in *Physcomitrella patens* as enhancers or repressors in the homologous recombination-mediated DSB repair (Kamisugi et al., 2012; Kamisugi et al., 2016; Wiedemann et al., 2018; Guyon-Debast et al., 2019).

The moss *Physcomitrella patens* belonging to the Funariaceae family of the Phylum Bryophyta is an increasingly popular model organism for studying plant evolution, development, and growth (Wood et al., 2000; Cove, 2005; Vidali et al., 2009). The Gransden strain of *Physcomitrella patens* was established in the United Kingdom and is now the most prevalent system used for genetic engineering in the laboratory (Engel, 1968). This non-vascular early land plant was sequenced in 2007 (Rensing et al., 2008), revealing a genome size of 511 Mb with 27 pseudochromosomes. A comparative genomic study indicated that *P. patens* shared a high degree of homology with high plants such as *A. thaliana* (Reski et al., 1998; Nishiyama et al., 2003). For example, more than 66% of *A. thaliana* proteins have homologs in *P. patens*. When combined with other advantages such as a completely sequenced genome, short life cycle, easy culture and maintenance, simple morphology, and polarized tip growing rhizoids and protonema, *Physcomitrella patens* becomes a powerful model system for the study of gene functions (Cove, 2005; Quatrano et al., 2007; Prigge and Bezanilla, 2010; Vidali and Bezanilla, 2012; Jaeger and Moody, 2021). More importantly, the predominant phase in the *P. patens* life cycle, from the germination of spores to the fertilization of eggs, is haploid, which allows screening of knockout mutant could complete in one generation.

## Generation of Successful Gene Knockouts by Gene Targeting

### Design of Knockout Constructs

**FIGURE 1:**
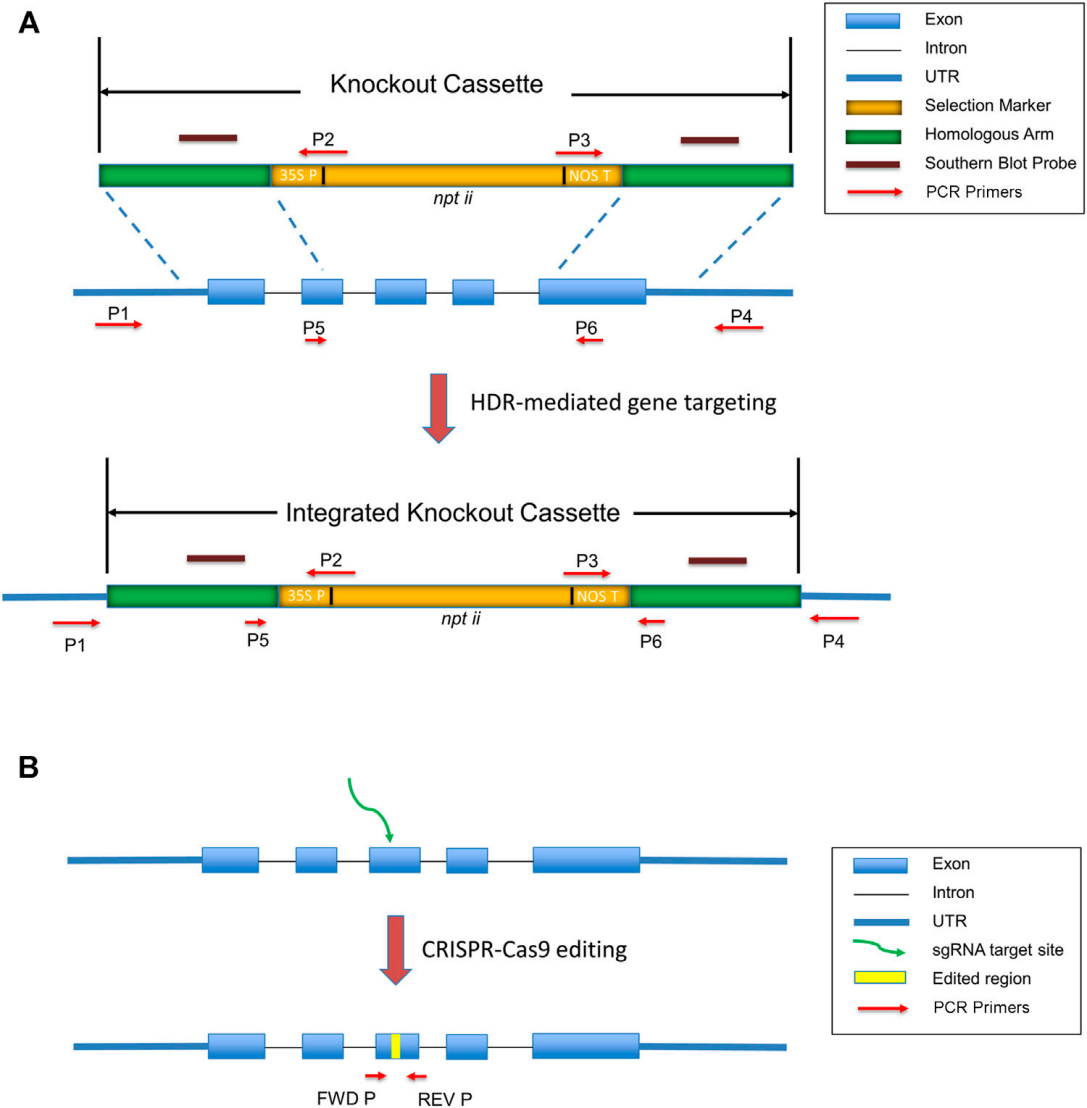
Targeted gene knockout generated by gene targeting and CRISPR-Cas9. **(A)** A successful gene targeting event results in targeted gene replacement. The knockout cassette contains homologous arms flanking *npt ii* genes driven by 35S promoter and tailed with a nos terminator. By transformation of a linearized knockout cassette to the moss protoplasts, the marker gene *nptii* is integrated into the genome, resulting in targeted gene replacement. **(B)** A frameshift mutant generated by CRISPR-Cas9. The yellow bar indicates gene-edited sequences. The green arrow suggests the target site. Red arrows show the location of primers (FWD P and REV P) for the amplification of the edited site.

To generate targeted knockouts efficiently, a good design of knockout cassettes is necessary. Typically, a knockout construct containing a selection marker flanked by left and right homologous arms comprising either cDNA or genomic sequences homologous to the target sites. Hygromycin or Geneticin resistant genes are common selection markers in the knockout cassettes (Schaefer and Zryd, 1997). Selection markers should insert into exons to ensure their expression in the regenerated plants. PHYSCObase provides a pre-assembled plasmid pTN80 (Accession: AB267704.2, G.I: 379,990,978) to the community to easily construct a knockout cassette. Figure 1A illustrates a knockout cassette prepared from pTN80, which comprises the *npt ii* gene as a selection marker driven by a 35S promoter and tailed with a nos terminator. Homologous arm sequences with equivalent length could target conserved domains, activation sites, promoters, or UTRs. 1 kb homologous sequences are sufficient to obtain gene knockouts, although 600–700 bp long homologous arms are also adequate to generate successful knockout transformants (Kamisugi et al., 2005). The length of homologous arms is correlated to the on-target insertions (Shy et al., 2016).

### Protoplast Transformation and Regeneration

Two decades ago, when gene targeting emerged, particle bombardment transformation to vegetative tissue or PEG-mediated transformation to protoplasts were both used by researchers (Cho et al., 1999; Bezanilla et al., 2003). Recently, the transformation of knockout cassettes to protoplasts has become prevalent in *Physcomitrella* gene targeting (Schaefer et al., 1991; Schaefer and Zryd, 1997; Rensing et al., 2020). PEG-mediated transformation and protoplast regeneration protocols are available on PHYSCObase and CSH protocol (Cove et al., 2009a). Moss protoplasts are filamentous protonema of which cell walls are degraded by Drislease (Grimsley et al., 1977). Both circular and linearized knockout constructs can use in the transformation. However, circular DNA may undergo extrachromosomal replication in transient knockouts and reduce the number of stable transformants. Therefore, linearized knockout cassettes are preferably transformed into protoplasts (Strepp et al., 1998; Ashton et al., 2000; Mittmann et al., 2004). Transformed protoplasts resuspended in PRMB medium incubate in the dark overnight and regenerate for 7 days under normal growth conditions. The two-round selection of stable transformants is carried out on the solid culture medium containing antibiotics for 2–3 weeks per round with a one- or 2-week interval on an antibiotic-free medium.

### Challenges and Technical Difficulties

Even though targeted gene knockouts mediated by homologous recombination are potent tools, there are challenges and fundamental issues with gene targeting. First, off-target events are the major challenge in gene targeting. The donor knockout cassettes transformed to the protoplasts integrate into the moss genome by either targeted gene replacement, targeted gene insertion, or illegitimate insertion (Kamisugi et al., 2005; Kamisugi et al., 2006). Among the three integrations, targeted gene replacement is ideal for integrating two homologous arms to the target loci by high-frequency homologous recombination in *Physcomitrella patens*. However, targeted insertions with one end on-target while the other end off-target is commonly present. Meanwhile, insertions of knockout cassettes randomly into the moss genome mediated by NHEJ might associate with targeted gene replacement. Additionally, researchers have found that multiple linearized knockout cassettes might form concatemers during the protoplast transformation (Kamisugi et al., 2006).

Validation of knockouts containing a single targeted gene replacement event is therefore essential before the phenotypic analysis. PCR is the primary method to confirm a single-copy integration of the exogenous knockout construct into the moss genome. As shown in Figure 1A, primer pairs P1/P2 and P3/P4 can amplify the insertion junctions of 5′ and 3’ ends, respectively. P5 and P6 are a pair of primers for the detection of RNA transcription to confirm the depletion of the targeted region. Southern blot furtherly confirms the single-copy insertion and excludes the ectopic insertions on the moss genome. The probes hybridize to the two homologous arms (Khandelwal et al., 2010).

Secondly, Protoplast fusion is present in the PEG-mediated transformation (Grimsley et al., 1977). The two broken protoplasts may fuse to make a diploid cell with one copy of the knockout allele and another copy of the wild-type allele during transformation and regeneration. For this case, PCR primers P1/P4 illustrated in Figure 1A can identify the wild-type gene copy in the knockouts.

Thirdly, the knockout of essential genes and multiple-gene families is challenging. Depletion of crucial genes often results in lethal mutants. Knockout multiple gene families or genes with redundancy are also tricky with gene targeting. Transformation of multiple knockouts constructs simultaneously to protoplasts may result in concatemers in the target loci.

## CRISPR-CAS9 Mediated Genome Editing

CRISPR-Cas9 system as an efficient genome editing tool, in which Cas9 cleaves DNA at target sites specified by guide RNA, has been applied to many plant species, including *Physcomitrella patens*. (Li et al., 2012; Shan et al., 2013; Rensing et al., 2020; Shan et al., 2020). Repair pathways of double-strand breaks (DSBs) in the traditional gene targeting and the CRISPR-Cas9 system are different. Gene targeting depends on homologous recombination, whereas Cas9-induced breaks are mainly repaired by NHEJ or alternative end-joining (Alt-EJ) (Collonnier et al., 2017). CRISPR/Cas9 can produce deletions, insertions, and substitutions, which could be frameshift mutants, early terminations, splicing variants, etc. Figure 1B shows a targeting site edited by CRISPR-Cas9 system.

To compare CRISPR-Cas9 and traditional gene targeting in detail, Table 1 lists aspects related to using two technologies in *Physcomitrella patens*, covering topics from vector construct, protoplast transformation and regeneration, selection of mutants, molecular analysis to mutant types, multiplexing options, and efficiency. Additionally, scientists have expanded the CRISPR-Cas9 toolkit to control gene editing tightly.

**TABLE 1:** **Comparison of the use of gene targeting and CRISPR-Cas9 in *Physcomitrella patens.*** Discussion topics cover constructs, protoplast transformation and regeneration, selection of knockouts, molecular analysis, mutant types, efficiency, and multiplexing knockouts.

	CRISPR-Cas9 Technology	Gene Targeting	References
Construct	sgRNA and Cas9 plasmids	Knockout cassette	Collonnier et al. (2017)
			Mallett et al. (2019)
			Girke et al. (1998)
Protoplast Transformation and Regeneration	PEG-mediated protoplast transformation	PEG-mediated protoplast transformation	Schaefer & Zryd (1997)
			Cove et al. (2009a)
			Radin et al. (2021)
Selection of Knockouts	Selection of regenerated plants harboring transiently expressed Cas9 and sgRNA;	Selection of regenerated plants comprising stable integration of knockout cassettes;	Cove et al. (2009b)
	1-week selection on antibiotics medium	Two rounds of selection on antibiotic medium with a 1-week interval	Hiwatashi & Hasebe (2012)
Molecular Analysis	PCR, T7 endonuclease assay,	PCR, RT-PCR, Southern blot	Steinberger et al. (2021)
			Gomann et al. (2021)
			Mallett et al. (2019)
			Khandelwal et al. (2010)
Mutant Types	Frameshift mutants (knockouts),	Gene knockouts,	Guyon-Debast et al. (2021)
	Base-edited mutants,	Knock-in mutant,	Radin et al. (2021);
	Marker-free mutants,	Marker gene or tag integrated to moss genome,	Brejskova et al. (2021)
	Loss-of-function or gain-of-function allele	Complementation line	Schaefer (2001)
Efficiency (targeted mutant/regenerated protoplasts)	2-3%	0.25%	Collonnier et al. (2017)
Multiplexing Knockouts	Simple, by the single transformation event	Time-consuming, usually by sequential transformations	Trogu et al. (2020)
			Lopez-Obando et al. (2016)
			Takechi et al. (2021)

### Construct of sgRNA and Cas9

Compared to the knockout cassette used in gene targeting, Cas9 and sgRNA are two components required for delivery to host cells in the CRISPR-Cas9 system (Collonnier et al., 2017; Mallett et al., 2019). Importantly, guide RNA specificity is critical for efficient editing through CRISPR-Cas9. To minimize the off-target events, specific gDNA sequences can be obtained in software called CRISPOR (Haeussler et al., 2016). Synthesized gDNA (sgDNA) and Cas9 are cloned to plasmids harboring additional selection marker genes that assist the transient selection of the regenerated protoplasts (Trogu et al., 2020; Yi and Goshima, 2020).

Promoters for the expression of Cas9 and sgRNA are studied. The most commonly used promoters include rice actin (Act) promoter and maize ubiquitin promoter for the expression of Cas9, and *Physcomitrella patens* U6 promoter for driving the expression of sgRNAs (Mallett et al., 2019). SgRNA and Cas9 plasmid should be at an equal ratio when transformed to protoplasts (Collonnier et al., 2017). Instead of separate plasmids for Cas9 and sgRNA, a modular CRISPR-Cas9 vector system has been developed to drive the expression of Cas9 and multiple sgRNAs simultaneously (Mallett et al., 2019). A successful application of this vector system was to generate seven mutant lines targeting the SBH gene (Steinberger et al., 2021). In the same vector system, up to 4 sgRNAs can be assembled with three choices of antibiotics resistant genes, hygromycin, G418, and zeocin, which enable targeting up to 12 genome sites in a single transformation.

### Protoplast Transformation and Regeneration

Delivery of Cas9 and sgRNA to plants could be achieved by Agrobacterium-mediated stable transformation. The transformation rate for protoplast transformation using Agrobacterium is typically 10^−4^, calculated by the number of stable transformants divided by surviving regenerants after transformation (Cove et al., 2009b). However, Agrobacterium-mediated transformation involves in the integration of exogenous DNA into target plants and the resulting mutants could be considered as genetically modified organisms. Instead, the protoplast transformation of plasmids that transiently express in plant cells will generate transgene-free knockout mutants. Although protoplast transformation and regeneration are a bottleneck and are currently developing in many plant species (Hsu et al., 2021; Li et al., 2021), PEG-mediated protoplast transformation has been widely used in P. patens study for gene targeting, including steps of cell wall degradation, protoplast resuspension, plasmid transformation, and protoplast regeneration and selection of knockouts on growth medium (Schaefer et al., 1991; Schaefer, 2001; Cove et al., 2009a). The same protocol can be utilized in the CRISPR-Cas9 system, except for different vectors for transformation (Lopez-Obando et al., 2016; Collonnier et al., 2017; Radin et al., 2021). 10–30 ug of Cas9 and sgRNA constructs are introduced to the 4.8 × 10^5^ resuspended protoplasts. Co-transformation of transiently expressed marker genes with Cas9 and sgRNA plasmids enables the reduction of false-positive clones (Gomann et al., 2021). Upon transformation, protoplasts grow on the cellophane overlaid on the regeneration medium for 4–7 days (Cove et al., 2009b; Radin et al., 2021).

### Selection of Knockouts

Knockout selection in CRISPR-Cas9 system can be completed in a week. Compared to the 1-month selection of stable knockouts by gene targeting, selection of transiently expressed Cas9 and sgRNA only takes 7 days. One round of selection against proper antibiotics is sufficient to confirm the Cas9 and sgRNA presence in the regenerated clones. This saves time to generate knockouts and increases the number of regenerated plants, as shown in a more significant number of clones emerging from the first-round selection than that from the 2nd round selection on the medium conferring antibiotic resistance (Hiwatashi and Hasebe, 2012).

### Molecular Analysis of Putative Knockouts

Whether in traditional gene targeting or CRISPR-Cas9 system, individual clones are sub-cultured on a standard growth medium for 2–3 weeks after selection. Protonema or young gametophytes are harvested to extract gDNA for molecular analysis of mutants. The transient expression of Cas9 and sgRNA in CRIPSR-Cas9 system allows for an increased number of regenerated plants. However, this requires an expanding screening work to identify edited plants from non-transgenic and non-edited surviving plants after transformation and regeneration.

For knockout screening, PCR amplification around the expected editing sites is a primary method to validate knockouts generated by gene targeting and CRISPR-Cas9. Additionally, unlike analysis methods such as RT-PCR and Southern blotting used in gene targeting, T7 endonuclease assay is utilized to screen potential knockouts in the CRISPR-Cas9 technique (Mallett et al., 2019). Screening of a large number of mutants can be performed by visualizing amplicons on 3% of agarose gel or the high-resolution PAGE gel (Lopez-Obando et al., 2016; Trogu et al., 2020). In this way, clones with base-pair changes can be detected on the gel and subsequently sequenced. Off-target events in CRISPR-Cas9 are also checked by amplifying potential off-target sites, but often no mutations on these putative off-target loci are detected (Collonnier et al., 2017).

### Mutant Types

Successful gene targeting events can generate both targeted gene knockouts and knock-in alleles at expected genomic sites. Those knockout or knock-in lines comprise selection markers or tags stably integrated into the genome (Schaefer, 2001). Furthermore, gene targeting can produce complementation lines to rescue the mutant phenotype by targeting full-length cDNA to the moss genome (Brejskova et al., 2021).

Knockouts generated by typical CRISPR-Cas9 resulted from NHEJ or Alt-NJ, harbor deletions, insertions, and rarely substitutions, most of which are frameshift mutations (Lopez-Obando et al., 2016; Mallett et al., 2019). Furthermore, in combination with donor DNA templates, CRISPR-Cas9 can also generate knock-in and gain-of-function alleles (Guyon-Debast et al., 2021; Radin et al., 2021). We will discuss this in the later section. Overall, CRISPR-Cas9 edited knockouts are free of the transgene.

### Knockout Efficiency

Traditional gene targeting is only limited to certain organisms such as mice, yeast, etc. Although the homologous recombination rate is much high in *P. patens*, obtaining successful knockout lines through homologous recombination is not as highly efficient as the CRISPR-Cas9 system. As shown in the literature, the traditional gene targeting only resulted in 0.25% relative transformation efficiency, whereas CRISPR-Cas9 reached 2.1–3.2% relative transformation efficiency from different sgRNAs using *PpAPT* as a target gene (Collonnier et al., 2017). That being said, for 15,000 regenerated clones, about 315 ∼ 480 knockouts survived in the CRISPR-Cas9 editing system, but only 37 knockouts emerged from traditional gene targeting.

### Multiplexing Knockouts

*Physcomitrella patens* have undergone genome duplications, and thus many gene families expanded to contain multiple genes (Zimmer et al., 2013; Li et al., 2015). Therefore, generation knockouts of multiple genes are necessary to study gene family functions. Both traditional gene targeting and the CRISPR-Cas9 system can generate multi-gene knockouts. However, multi-gene targeting using traditional gene targeting is time-consuming, associated with many rounds of selection (Takechi et al., 2021). In comparison, the generation of multi-gene knockouts using CRISPR-Cas9 is much easier and simpler. Co-transformation of Cas9 and multiple sgRNAs could result in multiplex gene knockouts. For example, a septuple knockout mutant was generated by a single transformation of Cas9 and sgRNAs (Trogu et al., 2020). Multiple genes from different gene families can also be targeted in the CRISPR-Cas9 system. Notably, single or double mutants may be present as byproducts during the transformation of multiple sgRNAs and Cas9 to moss protoplasts, creating genetic variations (Lopez-Obando et al., 2016).

### Editing *Physcomitrella patens* Genome Precisely and Efficiently

CRISPR-Cas9 is an efficient gene-editing system that results in deletions, insertions, and substitutions. Because *Physcomitrella patens* has a high frequency of homologous recombination, scientists aim to take advantage of this feature to control CRISPR-Cas9 editing tightly in the moss. Donor DNA templates are co-transformed with plasmids harboring Cas9 and sgRNAs to moss protoplasts. Collonnier reported that 60% of CRISPR-Cas9 induced DSBs were repaired *via* HDR, compared to 54% gene targeting efficiency shown in the same paper (Collonneir et al., 2017). Either single-strand (ss) DNA or double-strand (ds) DNA oligos, linearized plasmids, or circular plasmids can function as donor DNA templates (Collonneir et al., 2017; Mallett et al., 2019; Yi and Goshima, 2020; Radin et al., 2021). Consequently, DSB repair is tightly controlled by HDR, and genome editing in *P. patens* becomes more precise, resulting in 28–100% of colonies showing expected gene editing including substitutions, deletions, and knock-in tagging at target sites (Yi and Goshima, 2020). The donor DNA template-assisted or called oligodeoxynucleotide (ODN)-assisted CRISPR-Cas9 method generates knockouts, knock-in lines, and substitutions, which could be loss-of-function alleles, gain-of-function alleles, or hypoalleles that are beneficial for the study of essential genes.

Furtherly, a more precise mutation system called CRISPR-mediated base editors (BEs) has been developed in human murine cell lines, rice, and as well as in *Physcomitrella patens* (Komor et al., 2016; Li et al., 2017; Lu and Zhu, 2017; Guyon-Debast et al., 2021). In *P. patens*, CRISPR-Cas9 deaminase systems CBE was designed to obtain cytosine editing and ABE system was aimed for adenine editing in an predictable editing window, which was about -20 to -14 bp from PAM sites (Guyon-Debast et al., 2021). By co-transformation of sgRNAs specific to the reporter *PpAPT* gene driven by snRNA U6 promoter and nCas9 (D10A) fused with either *Petromyzon marinus* cytosine deaminase driven by pcUbi4-2 promoter or a heterodimer of wild-type and mutated *E. coli* tRNA adenosine deaminase driven by OsAct1 promoter, 89% mutants from a 2-FA selection corresponded to precise base editing through CBE system and 100% mutants survived on a 2-FA selection corresponded to A-to-G base editing in ABE system. For other targeting genes, CBE could result in up to 55% efficiency. The same study also showed that multiplex (up to 4 sgRNA targets) base editing was possible in *Physcomitrella patens* with CBE and ABE strategies.

## Conclusions

The moss *Physcomitrella patens* is a unique model organism harboring a high frequency of homologous recombination. Gene targeting has been widely used in *Physcomitrella patens* for the generation of targeted gene replacement. Recently, CRISPR-Cas9 technology is a hot topic in the genome-editing field. With its high efficiency and the resulting marker-free knockouts, CRISPR-Cas9 becomes increasingly appealing to scientists who work on *Physcomitrella patens*.
